# Property income *from-whom-to-whom* matrices: A dataset based on financial assets–liabilities stocks of financial instrument for Spain

**DOI:** 10.1016/j.dib.2018.05.018

**Published:** 2018-05-22

**Authors:** Luis Pedauga, Agustín Velázquez, Manuel Bueno

**Affiliations:** a University of León, Spain; b European Commission – Joint Research Centre (JRC), Spain; c University of Granada, Spain

## Abstract

A common problem in compiling and updating Social Accounting Matrices (SAM) or Input-Output tables is that of incomplete information. In the case of the submatrix ‘Property Income of the Account Allocation of Primary Income’, the information published by the National Bureau of Statistics of Spain (INE) is limited because it is not possible to build the set of *from-whom-to-whom* sub-matrix on income interest, dividends, securities and rents with only the subtotals presented in the Integrated Economic Accounts (IEA). This because the income distribution received and paid for by each institutional sector required for a financial SAM is not available, i.e. the INE does not break down the data by institutional destination and source. In this sense, our contribution rely on estimating a complete series of *from-whom-to-whom* matrices of Property Income for the Spanish economy between 1999 and 2016, in which we have devoted special attention to staying in line with the Data Gaps Initiative (DGI-2) recommendation released by the Financial Stability Board (FSB) and the International Monetary Fund (IMF), claiming that more focus is needed on data sets that support the monitoring of risks in the financial sector in response to regulatory and macro-financial emerging policy needs).

**Specifications Table**

**Table:** 

Subject area	*Economics*
More specific subject area	*Financial Macroeconomic, Flow of Funds, System of National Accounts*
Type of data	*Table, Excel-file*
How data was acquired	The information concerning the property income was retrieved from the Account Allocation of Primary Income from the National Bureau of Statistics of Spain. The information of the financial assets/liabilities statistics of the Flow of Funds (FoF) was retrieved from the Bank of Spain.
Data format	Raw, estimated
Experimental factors	The breakdown of the dataset was derived by applying a GRAS algorithm
Experimental features	Data included the balanced series of five type of financial transactions in a from-whom-to-whom scheme: (D.41) Interest income, (D.42) Distributed income of corporations, (D.43) Reinvested earnings on foreign direct investment, (D.44) Investment income attributable to insurance policyholders and (D.45) Rents. Additionally, include the stock of four financial instruments: (AF.2+AF.3+AF.4) Deposits, Securities and Loans, (AF.5) Shares and other equity, (AF.6) Insurance technical reserves and (AF.1+AF.7+AF.8) Other instruments.
Data source location	Spain
Data accessibility	Data is with this article

**Value of the data**

• The dataset provides accurate estimates of the allocation of primary income account – property income – in a *from-whom-to-whom* matrix scheme. This representation allows the question ‘who’ is financing ‘whom’ to be answered, which allows a more detailed and complex analysis of the financial flows between sectors and their role in the economy.
• The novel approach to provide the stocks of Asset and Liabilities Matrices in a *from-whom-to-whom* framework by financial instruments turns out to be very useful for analyzing the real-financial interconnectedness of the Spanish economy.
• The dataset provides the necessary elements to estimate the breakout of the total income return, resulting in an outstanding sources of information for investment analysis and impact analysis of public policies.
• The set of submatrices results in a consistent accounting framework useful for improving and extending Social Accounting Matrices, sectorial-financial linkage analysis, macroeconomic forecasting and improve and enrich the scope of real-financial computable general equilibrium (CGE) models.

## 1 Data

The real side information concerning the allocation of primary income account – property income – was obtained from the statistics of the Integrated Economic Accounts (IEA) provided by the National Bureau of Statistics of Spain (INE). The financial side information was retrieved from the financial statistics of the Flow of Funds (FoF) provided by the Bank of Spain (BdE). Both INE and BdE data sets correspond to the yearly series 1999 to 2016 due to the constraints to using the more recent official data set available to build a Property Incomes matrix for Spain. Given that both the real statistics of the INE and the financial statistics of the BdE shape the entire System of National Accounts (SNA93) [1], the estimation procedure proposed in this data research maintain and respect the statistical data provided by both agencies. In this sense, the statistical compilation procedures follow the UN Manual of SNA93 for the construction of the matrices of Property Incomes, while contemplating the recommendations made by Shrestha et al. [2] to expand the statistical information within an integrated framework for financial stocks positions and flows on a *from-whom-to-whom* basis, and the compilation guides suggested by Tsujimura and Mizoshita [3] and Jellema et al. [4] to integrate the financial matrices accounting used as baseline.

The statistical information from the INE is available in integrated structured tables separated by years, while the database from the BdE is expressed in quarterly time series. Since the data from the BdE are in quarterly series, different treatments among figures expressed in flows from those expressed in balances were required, adding the former to form flows for the year and considering the figures of the last quarter as the closing balances of the respective year.

## 2 Experimental design, materials and methods

In the wake of the 2008 financial and economic crisis, the Group of Twenty economies (G-20) asked the Financial Stability Board (FSB) and the International Monetary Fund (IMF) “to explore gaps and provide appropriate proposals for strengthening data collection before the next meeting of the G-20 Finance Ministers and Central Bank Governors.” In its Spring Meeting in April 2009, the FSB-IMF came up with 20 recommendations [5], known now as the Data Gaps Initiative (DGI-1), to address information gaps revealed by the global financial crisis. Recently, the FSB-IMF concluded the Second Phase of the G-20 Data Gaps Initiative (DGI-2) in September 2017 [6]. The DGI-2 recommendations maintain the continuity of DGI-1 but claim that more focus is needed on data sets that support the monitoring of risks in the financial sector and the analysis of the interlinkages across economic and financial systems. This data article focuses on two of these recommendations, both of which state that G-20 member economies should extend their national accounts by compiling financial and nonfinancial stocks and flows in the economic sector.

### 2.1 Integrated approach for property income and financial instruments on a *from-whom-to-whom* basis

The integrated system of sector accounts in a *from-whom-to-whom* (or debtor/creditor) framework, correspond to the matrix form representation that allow the analysis of the financial connections among institutional sectors in a national economy and abroad. As have been pointed out by Shrestha et al. [2] the integrated *from-whom-to-whom* representation of statistical information allows answering questions like “Who is financing whom, in what amount, and with which type of financial instrument?”. As regards of property income, it also permits tracing who is paying/receiving income (e.g., interest) to/from whom. The *from-whom-to-whom* compilation approach also enhances the quality and consistency of data by providing more cross-checking and balancing opportunities.

The System of National Accounts 2008 (SNA2008) [7] presents *from-whom-to-whom* matrices as three dimensional tables where the flows from one sector to another sector for each type of financial instruments are showed. In this regard, to estimate the matrix Property Income, we based our approach on the definition provided by the SNA2008 manual, which states:

“7.107 Property income accrues when the owners of financial assets and natural resources put them at the disposal of other institutional units. The income payable for the use of financial assets is called investment income while that payable for the use of a natural resource is called rent. Property income is the sum of investment income and rent.

7.108 Investment income is the income receivable by the owner of a financial asset in return for providing funds to another institutional unit…”.

Hence, we can use the balances account of the financial account relating to financial assets and liabilities across the institutional sectors as estimators of the shares of income received and paid by each institutional sector. Intuition suggests that the property income received and/or paid for each institutional sector should be directly proportional to its levels of assets and/or liabilities. Thus, we used the balance account of the financial account relating to financial assets and liabilities across the institutional sectors as estimators of the shares of income received and paid by each of them.

### 2.2 Property income *from-whom-to-whom* matrix

The property income *from-whom-to-whom* matrix show how the income is received by the owner of a financial asset in return for providing funds to another institutional sector. The income payable for the use of financial assets is called investment income while that payable for the use of a natural resource is called rent [7]. Formally, the property income as the total sum of investment income and rent, can be expressed as follow:

(1) $${\mathrm{PI}}_{\mathrm{mxmxp}}=\left(\begin{array}{ccc} x_{1x1\mathrm{xp}} & \cdots & x_{1\mathrm{xmxp}}\\ \vdots & \ddots & \vdots \\ x_{\mathrm{mx}1\mathrm{xp}} & \cdots & x_{\mathrm{mxmxp}} \end{array}\right)$$

where ${\mathrm{PI}}_{\mathrm{mxmxp}}$ correspond to the Property income matrix in a double and quadruple entry matrix form, the subscript m denotes institutional sectors in the economy, and the subscript p denotes income type of transactions. In this data article, we consider m equal to 5 institutional sectors,^1^ the main groups of economic agents who are responsible for changes and movements in National Accounts: (S.11) Non-financial institution, (S.12) Financial corporations, (S.13) General Government, (S.1M) Householders and Non-profit institutions serving householders (NPISHs), and (S.2) Rest of the world. Similarly, we consider p equal to 5 types of financial transactions: (D.41) Interest income, (D.42) Distributed income of corporations, (D.43) Reinvested earnings on foreign direct investment, (D.44) Investment income attributable to insurance policyholders and (D.45) Rents (see Table 1 for more details).

**Table 1:** Property income and type of payments.

**Property income**		**Type of payments**	
*Code*	*Description*	*Variable*	*Description*
D41	– Interest payable on loans and deposits.	Interest	Income receivable by the owners of certain kind of financial assets at the disposal of another institutional unit.
	– Interest payable on debt securities.		
D42	– Distributed income of corporations.	Dividends	Investment incomes to which shareholders become entitled as a result of placing funds at the disposal of corporations.
	– Withdrawals from incomes of quasi-corporations, Investment funds shareholders.		
D43	– Reinvested earnings of foreign direct investment.		
D44	– Income payable on pension entitlements, insurance policyholders.	Securities	Other investments incomes and rents.

From expression (1) we can get from each row the total vector $v_{\mathrm{jp}}$ of total property income paid by each m institutional sector:

(2) $$\mathrm{Paid:}\sum_{i}x_{\mathrm{ijp}}=v_{\mathrm{jp}}$$

Similarly, from each column the total vectors $u_{\mathrm{jp}}$ of total property income received by each m institutional sector:

(3) $$\mathrm{Received:}\sum_{j}x_{\mathrm{ijp}}=u_{\mathrm{ip}}$$

In this sense, the integrated framework on a *from-whom-to-whom* scheme allows answering questions like “Who is paying/receiving income (e.g., interest) to/from whom, in what amount, and with which type of transaction?”. Also, as have been pointed out by Shrestha et al. [2] this matrix representation approach enhances the quality and consistency of data by providing more cross-checking and balancing requirements, given that the following condition should be hold

(4) $$\mathrm{Equilibrium:}\sum_{\mathrm{ip}}u_{\mathrm{ip}}=\sum_{\mathrm{jp}}v_{\mathrm{jp}}$$

where the total paid must be equal to the total received by the economy.

### 2.3 Financial Instruments *from-whom-to-whom* matrices

Financial instruments include the full range of financial contracts made between institutional sectors. These contracts are the basis of creditor/debtor relationships through which asset owners acquire unconditional claims on economic resources of other institutional sectors [7]. In this sense, the financial instrument matrix defined as $A_{\mathrm{mxmxq}}$ denotes a *from-whom-to-whom* representation of the net worth of this economy in terms of stocks:

(5) $$A_{\mathrm{mxmxq}}=\left(\begin{array}{ccc} a_{1x1\mathrm{xq}} & \cdots & a_{1\mathrm{xmxq}}\\ \vdots & \ddots & \vdots \\ a_{\mathrm{mx}1\mathrm{xq}} & \cdots & a_{\mathrm{mxmxq}} \end{array}\right)$$

where $A_{\mathrm{mxmxp}}$ correspond to the Assets–Liabilities matrix of Stocks of Financial Instrument in a double and quadruple matrix form. The financial instrument *from-whom-to-whom* matrices in expression (5) comprise the financial acquisition in both claims (described as assets) and obligations (described as liabilities) by institutional sector. As before, the subscript m denotes institutional sectors in the economy, and the subscript q denotes financial instruments. The availability of information provided by the Bank of Spain allow to consider seven (q=7) financial instruments: AF.1 Monetary gold and Special Drawing Rights, AF.2 Currency and deposits, AF.3 Debt securities, AF.4 Loans, AF.5 Equity and investment fund shares, AF.6 Insurance, pension, and standardized guarantee schemes, and AF.7/8 Other Assets (see Table 2 for more details).

**Table 2:** Financial Instruments and type of assets.

**Financial Instrument**		**Type of asset/liability**	
*Code*	*Description*	*Variable*	*Description*
AF.1	– Gold and reserves. – Special Drawing rights.	Monetary gold and special Drawing rights.	Titles held as a reserve assets, comprising gold bullion and supplement reserve only produced by IFM.
AF.2	– Currency, Notes and coins issued or authorized by central bank or government. – Transferable deposits, Other deposits.	Currency and Deposits.	Amount of money in national or foreign currency that economic agents own as assets and liabilities. Saving deposits, fixed-term deposits and non-negotiable certificates of deposits.
AF.3	– Short-term and long-term securities.	Debt securities.	Negotiable instruments that can serve as evidence of a debt.
AF.4	– Short-term and long-term Loan.	Loans.	Financial assets created when a creditor lends fund directly to a debtor and evidenced by a document that they are not negotiable.
AF.5	– Listed and investment fund shares. – Unlisted and other Equity shares.	Equity and investments fund share.	Assets whit the particular feature that their holders obtain a residual claim of the institutional unit who issued the instrument.
AF.6	– Life and non-life insurance. – Pension entitlements.	Insurance, pensions and standardized guarantees.	All function as a form of redistribution of income or wealth mediated by financial institution.
AF.7/8	– Financial derivatives. – Trade credits, other accounts receivable and advances.	Other assets.	All other kind of financial assets linked to a specific financial instrument or destined to goods and services.

### 2.4 GRAS estimation approach

Like Leung and Secrieru [8] and Aray, Pedauga and Velázquez [9] we estimated the Property Income matrix (${\mathrm{PI}}_{\mathrm{mxmxq}}$) breakdown by institutional sector and type of instruments defined in Eq. (1) by using the information embedded on the assets and liabilities matrix of each institutional sector compiled in the financial instruments matrix ($A_{\mathrm{mxmxq}}$) represented in Eq. (2). In this sense, let A and PI be, respectively, the observed (prior) and the estimated (target) matrix, with their typical elements $a_{\mathrm{ijq}}$ (each financial instrument) and $x_{\mathrm{ijp}}$ (each property income component).

Under the GRAS algorithm [10], the prior matrix A is used as baseline to estimate the target matrix PI, satisfying simultaneously the row sums $v_{\mathrm{jp}}$ defined in Eq. (2) and column sums $u_{\mathrm{jp}}$ expressed in Eq. (3). Thus, the programming model following the information loss problem is such that:

(6) $$\min\sum_{i,j}\left[\left|\alpha_{i}{\cdot a}_{\mathrm{ijq}}\cdot \beta_{j}\right|\left(\frac{x_{\mathrm{ijp}}}{a_{\mathrm{ijq}}}\right)\cdot \log\left(\frac{x_{\mathrm{ijp}}}{a_{\mathrm{ijq}}}-1\right)\right]$$

(7) $$\mathrm{subject}\;\mathrm{to:}\sum_{j}x_{\mathrm{ijp}}=u_{\mathrm{ip}}\mathrm{for}\;\mathrm{all}\;i,\;$$

(8) $$\sum_{i}x_{\mathrm{ijp}}=\;v_{\mathrm{jp}}\mathrm{forall}j,\mathrm{and}$$

(9) $$\alpha_{i}=\{\begin{array}{c} 0\;\forall \;u_{\mathrm{ip}}=0\\ 1\;\forall \;u_{\mathrm{ip}}\neq 0 \end{array}\;\beta_{j}=\{\begin{array}{c} 0\;\forall \;v_{\mathrm{jp}}=0\\ 1\;\forall \;v_{\mathrm{jp}}\neq 0 \end{array}$$

Eq. (6) represents the objective function. Constraints (7) and (8) imply that the adjusted matrix PI should be consistent with an exogenously specified row and column totals. Moreover, constraint (9) introduces parameters $\alpha_{i}$ and $\beta_{j}$ which make all cells 0 in a row i or a column j of matrix PI, when the corresponding cell in $u_{\mathrm{ip}}$ or $v_{\mathrm{jp}}$ is 0. These new constraint is set to remove Financial Assets/Liabilities in matrix A which do not produce payments in matrix PI.

In this sense, we are capable to derive the breakdown of the Property Income by types of financial transactions for the Spanish economy, in which we have devoted special attention to staying in line with DGI-2 recommendation II.8, referring to the compilation of sectorial account flows and balance sheet data, based on from-whom-to-whom matrices expressed in transactions and stocks to support balance sheet analysis, and recommendation II.9, which encourages the development and dissemination of distributional information on income allocation.

## Footnotes

1 An important feature to be considered is that the SNA uses various information classifiers contained in the INE and BdE statistics. These information classifiers were maintained and used to present the dataset.
